# Falling into a burning ring of fire: A case of psychosis unmasking hidden neurosyphilis

**DOI:** 10.1192/j.eurpsy.2021.1428

**Published:** 2021-08-13

**Authors:** S. Jesus, A. Costa, J. Alcafache, P. Garrido

**Affiliations:** Psiquiatria E Saúde Mental, Centro Hospitalar do Baixo Vouga, Aveiro, Portugal

**Keywords:** psychosis, Neurosyphilis, differential diagnosis

## Abstract

**Introduction:**

The first description of syphilis was made in Europe around the year 1493, and although perceived as a disease relegated to its historical importance, recent studies demonstrate that the prevalence of these infections is on the rise. Spanning decades after initial infection, 30% of affected individuals without treatment may develop tertiary syphilis, which includes neurosyphilis. Its notoriously “chameleon-like” presentation implies the necessity to not overlook neurosyphilis as a differential diagnosis in psychiatric settings.

**Objectives:**

Case report study and discussion.

**Methods:**

The authors present a case of affective and psychotic symptoms (including auditory and visual hallucinations and persecutory delusions) of rapid onset in a 61-year old woman without prior psychiatric history. A clinical investigation was conducted, which subsequently revealed a positive Venereal Disease Research Laboratories (VDRL) test. A lumbar puncture was performed and cerebrospinal fluid analysis confirmed neurosyphilis.

**Results:**

Steady improvements in initial psychopathological manifestations were noted after completing recommended treatment for neurosyphilis. After discharge, the patient was medicated with an antidepressant and antipsychotic, demonstrating a complete return to baseline mentation and functionality on follow-up.

**Conclusions:**

This case demonstrates the vital importance of considering syphilis in our differentials, especially due to the wide range of manifesting psychiatric symptoms. Although considered a disease of the past, this case reminds us that syphilis remains present in our society and its timely diagnosis and treatment can ameliorate the debilitating psychopathological manifestations of the disease. Due to the potential difficulties in identifying this great imitator, routine screening tests are still recommended in the psychiatric setting.

